# Intraoperative Ischemia Threshold and Outcomes of Emergency Vascular Repair During Orthopaedic Arthroplasty: A Time-Critical Analysis from a Dedicated On-Call Vascular Service

**DOI:** 10.3390/jcm15135229

**Published:** 2026-07-04

**Authors:** Luca Galassi, Chiara Barillà, Federica Facchinetti, Carlo Banfi, Filippo Benedetto

**Affiliations:** 1 Postgraduate School of Vascular and Endovascular Surgery, University of Milan, Via Festa del Perdono 7, 20122 Milan, Italy; 2 Vascular and Endovascular Surgery Unit, IRCCS Ospedale Galeazzi—Sant’Ambrogio, Via Cristina Belgioioso 173, 20157 Milan, Italy; 3 Vascular Surgery Unit, Department of Biomedical, Dental and Morphological and Functional Imaging Sciences, University of Messina, Via Consolare Valeria 1, 98125 Messina, Italy; chiara.barilla@unime.it (C.B.); filippo.benedetto@unime.it (F.B.); 4 School of Medicine and Surgery, University of Milan-Bicocca, Via GB Pergolesi, 20900 Monza, Italy; f.facchinetti4@campus.unimib.it; 5 Geneva Hemodynamic Research Group, University of Geneva, 1211 Geneva, Switzerland; carlo.banfi@unige.ch

**Keywords:** vascular injury, orthopaedic surgery, intraoperative complications, limb salvage, emergency vascular surgery, arthroplasty, ischemia time, on-call surgery, Rutherford classification

## Abstract

**Background**: Intraoperative vascular injuries during elective hip and knee arthroplasty are uncommon but limb-threatening complications. Real-world evidence on emergency on-call vascular management in this setting remains limited. We aimed to identify the intraoperative ischemia time threshold associated with progression to a more severe ischemic presentation (Rutherford IIb) at vascular consultation, in order to support early multidisciplinary activation and prevent irreversible ischemic limb damage. As a secondary aim, we described the clinical spectrum, treatment strategies, and 30-day outcomes of patients managed by a 24 h on-call vascular service (in-hospital coverage during working hours, formal on-call rota out of hours). Non-ischaemic events recorded in the series (e.g., isolated venous injuries and haemorrhagic complications) are documented as part of the overall clinical spectrum but were not the subject of specific time-related analysis. **Methods**: Single-centre retrospective analysis of 33 consecutive patients undergoing emergency vascular intervention for vascular injury during elective total knee (TKA) or total hip arthroplasty (THA) at a tertiary orthopaedic referral centre in Milan, Italy (January 2023—December 2025). The primary analytical objective was to identify the intraoperative ischemia time threshold associated with Rutherford IIb presentation at vascular consultation; 30-day limb salvage was the primary clinical outcome. Secondary outcomes included technical success, primary 30-day patency, postoperative ankle–brachial index (ABI), length of stay, and Clavien–Dindo complications. Non-ischaemic events (including isolated venous injuries and haemorrhagic complications) are documented as part of the clinical spectrum but were not subject to specific time-related analysis. Receiver operating characteristic (ROC) analysis assessed the discriminative role of intraoperative ischemia time for a Rutherford IIb presentation; univariate logistic regression explored predictors of postoperative complications. **Results**: Thirty-three patients (mean age 76.3 ± 6.3 years; 54.5% female; ≥2 comorbidities in 81.8%) underwent emergency vascular repair after TKA (60.6%) or THA (39.4%). Injuries were mixed arteriovenous (54.5%), purely venous (24.2%), or purely arterial (21.2%). Mean call-to-incision time was 45.4 ± 11.3 min. In the 25 ischemic cases, the mean intraoperative ischemia time was 130.4 ± 18.7 min. ROC analysis identified an optimal cut-off of 131 min for Rutherford IIb (AUC 0.851, 95% CI 0.679–0.982; *p* < 0.001), with sensitivity 81.8% and specificity 85.7%. Median ischemia time was significantly higher in IIb than IIa cases (144 vs. 124.5 min; *p* = 0.003). Technical success and 30-day limb salvage were 100% (95% CI 89.6–100); mean postoperative ABI 0.89 ± 0.03; primary 30-day patency 88.0% (95% CI 70.0–95.8), with secondary patency 100%. All postoperative complications were Clavien–Dindo grade 1; no Clavien–Dindo ≥ 2 events and no 30-day mortality were observed. **Conclusions**: A dedicated 24 h on-call vascular service achieves excellent 30-day limb salvage and patency in iatrogenic vascular injuries occurring during arthroplasty. An intraoperative ischemia threshold of 131 min identifies higher-risk presentations and supports rapid multidisciplinary activation in high-volume orthopaedic centres.

## 1. Introduction

Total knee arthroplasty (TKA) and total hip arthroplasty (THA) are among the most frequently performed elective procedures in adult orthopaedic surgery. In the United States, the annual volume of primary TKA approached half a million procedures by 2019, with contemporary projections estimating an increase to approximately 1.26 million procedures per year by 2030 [1] and further substantial growth by 2040 [2], in parallel with population ageing and the rising prevalence of obesity. Comparable volume trends are observed across most European registries, including in Italy, where elective hip and knee replacements remain among the highest-volume orthopaedic procedures.

Direct iatrogenic vascular injury during these operations is rare but far from negligible. The most comprehensive meta-analysis available, pooling data from over 1.4 million TKA, identified 767 major vascular events (0.05%); around one-fifth of patients with major injury required amputation or developed long-term neurological sequelae, and more than a third of injuries were diagnosed only beyond the first 24 h after surgery [3,4]. A more recent systematic review summarised an aggregate vascular complication rate of approximately 0.3%, dominated by ischemia and thrombosis, pseudoaneurysms, and frank haemorrhage or transection [5]. Around the hip, pooled estimates set the incidence of vascular injury between 0.16% and 0.25%, with the external iliac and common femoral vessels most often involved and a documented mortality of up to 7% in older series [6]. Population-based national data on popliteal artery injury during knee replacement showed an incidence of 0.017%, with amputation in 21% and a similar mortality figure [7].

Mechanisms of injury differ between the two procedures. At the knee, posterior retraction during the tibial cut, instrumentation of the femoral notch, and tourniquet effects on calcified vessels are typical contributors to popliteal or superficial femoral artery damage [3,8]. At the hip, retractor compression of the iliac axis, acetabular reaming, intrapelvic protrusion of cement or screws, and cerclage wiring during revision can injure both the iliofemoral arteries and the venous compartment [6]. Although laceration is the classic lesion seen at THA, contemporary series increasingly report mixed thrombotic and embolic patterns, often appreciated only after restoration of perfusion or at the time of distal pulse re-examination [5,9].

Time-to-revascularization remains the single most influential determinant of outcome in iatrogenic acute limb ischemia (ALI). The European Society for Vascular Surgery (ESVS) 2020 guidelines on ALI recommend revascularization within six hours for Rutherford IIb presentations, with progressively worse outcomes thereafter [10,11]. The 2025 ESVS Vascular Trauma guideline reinforced the requirement for a continuously available 24 h vascular service, capable of both open and endovascular repair, in any centre performing high volumes of orthopaedic surgery [12]. Nonetheless, much of the published experience on intraoperative arthroplasty-related vascular complications still comes from heterogeneous, multi-decade case series in which transfer to a vascular centre frequently delays revascularization by several hours [5,9,13].

Few studies have specifically addressed the modern paradigm of single-institution, on-call vascular intervention activated within the same operating session. Furthermore, no study to date has quantified the intraoperative ischemia threshold beyond which patients are more likely to present with a more severe Rutherford class at the moment of vascular consultation, despite the obvious relevance of such an indicator for early activation protocols. The primary aim of the present study was to identify the intraoperative ischemia time threshold beyond which patients are more likely to progress to a more severe ischemic class (Rutherford IIb) at vascular consultation, providing a quantitative reference for early activation protocols and the prevention of ischemic limb damage. The secondary aim was to describe the clinical spectrum, treatment strategies, and 30-day outcomes of consecutive patients managed by a dedicated 24 h on-call vascular service for intraoperative injury during TKA or THA at a single tertiary orthopaedic centre. Non-ischaemic events recorded in the series, including isolated venous injuries and haemorrhagic complications, are documented as part of the overall clinical spectrum but were not subject to specific time-related analysis.

## 2. Materials and Methods

### 2.1. Study Design and Setting

We conducted a single-centre, retrospective, observational cohort study of consecutive patients who required emergency on-call vascular intervention for vascular injury occurring during elective TKA or THA at IRCCS Ospedale Galeazzi-Sant’Ambrogio (Milan, Italy) between 1 January 2023 and 31 December 2025. The institution is a high-volume orthopaedic referral centre with a dedicated vascular surgery service available on a 24 h basis: vascular surgeons provide in-hospital coverage during working hours and continue to ensure continuous availability outside working hours through a formal on-call rota with a contractual response time. All vascular activations are prospectively logged in the institutional vascular emergency registry; clinical, operative, and follow-up data were retrieved from the electronic medical record. The study was reported in accordance with the Strengthening the Reporting of Observational Studies in Epidemiology (STROBE) statement [14] (see Table S1). 

### 2.2. Ethical Considerations

The study was conducted in accordance with the principles of the Declaration of Helsinki of 1975, as revised in 2013. Clinical data were extracted in fully de-identified form from the institutional vascular emergency registry as part of standard care; no modification of clinical management and no additional intervention on the patients were undertaken for the purpose of this analysis. At the time of admission, all patients had provided written consent for the use of their de-identified clinical data for scientific research purposes, in line with institutional policy. Under the current Italian regulatory framework on the processing of personal data for scientific research, Article 110 of Legislative Decree no. 196/2003, as amended by Legislative Decree no. 101/2018 implementing Regulation (EU) 2016/679 (General Data Protection Regulation), together with the General Authorisation of the Italian Data Protection Authority on the processing of personal data for scientific research purposes (Provvedimento del Garante no. 146 of 5 June 2019), secondary, retrospective, non-interventional research conducted on pre-existing, anonymised clinical data of this kind does not require formal ethics committee review and approval. All data were processed in compliance with these provisions and with the institutional data protection policy.

### 2.3. Inclusion and Exclusion Criteria

All consecutive adult patients (≥18 years) undergoing primary or revision TKA/THA in whom an arterial, venous, or combined vascular injury was identified intraoperatively and managed by the on-call vascular team during the study period were included. Exclusion criteria comprised:

(i) Delayed vascular presentation beyond 24 h from the index orthopaedic procedure.
(ii) Vascular injuries unrelated to the arthroplasty (for example, central line placement complications).
(iii) Incomplete operative records. The study time frame coincided with the consolidation of the institutional on-call vascular activation protocol described below. No patient who met the inclusion criteria was lost to follow-up at 30 days.

### 2.4. On-Call Vascular Activation Protocol

When unexpected bleeding, loss of distal pulse, or intraoperative anatomical concern is recognised by the orthopaedic team, the vascular service is activated through a single-call alert. The standard protocol provides for the arrival of the consultant vascular surgeon in the operating room within 30 min from notification, immediate transfer from ongoing in-hospital duties during working hours, and within the contractual on-call response window outside working hours, immediate availability of an endovascular-capable hybrid theatre on the same campus, and on-table consultation with the orthopaedic surgeon. Initial vascular evaluation includes inspection of the operative field, palpation and continuous-wave Doppler assessment of distal pulses, intraoperative ankle–brachial index measurement when feasible, and selective intraoperative angiography in arterial cases. The decision between open, endovascular, or hybrid approach is taken jointly with the orthopaedic team, considering the lesion pattern, the vessel involved, prosthetic constraints, and patient haemodynamic stability.

### 2.5. Variables and Definitions

Demographic data, comorbidities (diabetes mellitus, coronary artery disease, chronic kidney disease, atrial fibrillation, active smoking, and arterial hypertension), and surgical parameters were collected through a structured datasheet by two investigators independently and cross-checked for consistency. Vascular injuries were classified according to type (arterial, venous, mixed arteriovenous), pattern (laceration, occlusion, compression, compression with kinking), vessel involved, mechanism (retractor traction, direct injury, instrumentation, clamp-related), and underlying pathophysiology (thrombosis, embolism, bleeding).

Two time intervals were prospectively recorded. Call-to-incision time was defined as the interval, in minutes, from the moment of vascular team activation to the first vascular incision (or arterial puncture in purely endovascular procedures). Ischemia time, applicable to arterial and mixed cases only, was defined as the interval from documented vascular flow disruption (loss of distal pulse, arterial occlusion at angiography, or first description of ischemic findings in the operative note) to restoration of inline flow. Ischemia time was conventionally set to zero in purely venous lesions without arterial compromise.

Severity of acute limb ischemia at vascular consultation was graded according to the Rutherford classification [15]. Postoperative complications were graded with the Clavien–Dindo classification [16,17]. Technical success was defined as completion of the planned vascular procedure with restoration of vessel patency, absence of significant residual stenosis, and no contrast extravasation on completion angiography or duplex ultrasound. Limb salvage was defined as freedom from major amputation (above the ankle) at 30 days. Primary 30-day patency was assessed by clinical examination and duplex ultrasonography (or computed tomography angiography when clinically indicated) in all arterial and mixed cases. Secondary patency refers to vessel patency maintained by reintervention. Postoperative ankle–brachial index (ABI) was measured on the operated limb prior to discharge in arterial and mixed cases, with the limitations of resting ABI in elderly diabetic patients duly acknowledged [18].

### 2.6. Outcomes

The primary analytical objective was to define an intraoperative ischemia time threshold associated with Rutherford IIb presentation at vascular consultation. The primary clinical outcome was 30-day limb salvage. Secondary outcomes included technical success, primary and secondary 30-day patency, reintervention rate, postoperative ABI, length of in-hospital stay, intensive-care utilisation, and any postoperative complication graded according to Clavien–Dindo classification. An exploratory secondary objective was to investigate univariate predictors of postoperative complications. Non-ischaemic complications (including haemorrhagic events and isolated venous injuries) are documented as part of the clinical context but were not subject to specific time-related analysis.

### 2.7. Statistical Analysis

Continuous variables are presented as mean ± standard deviation (SD) or as median with interquartile range (IQR), depending on distribution. Distribution normality was tested with the Shapiro–Wilk test. Categorical variables are expressed as counts and percentages, with 95% confidence intervals (CI) calculated for key proportions using the Wilson score method. Comparisons between independent groups were performed with the Mann–Whitney U test for continuous data, chosen a priori in view of the limited sample size of the ischemic subgroup (*n* = 25), in which rank-based inference is more robust than parametric testing and is consistent with the rank-based nature of the ROC analysis. The rank–biserial correlation was reported as a non-parametric effect size.

To explore the discriminative ability of intraoperative ischemia time for the development of a Rutherford IIb presentation at vascular consultation, receiver operating characteristic (ROC) curve analysis was performed in the subset of arterial and mixed cases. The area under the curve (AUC) was reported with 95% confidence intervals calculated by 2000-iteration bootstrap resampling. The optimal cut-off was identified by maximisation of the Youden index (J = sensitivity + specificity − 1). The null hypothesis AUC = 0.5 was tested using a Hanley–McNeil-based standard error.

Univariate logistic regression was used to explore associations between candidate predictors (intraoperative ischemia time, Rutherford IIb class, age, body mass index, sex, presence of diabetes mellitus, and call-to-incision time) and the occurrence of any postoperative complication (Clavien–Dindo grade ≥ 1). Effect estimates are reported as odds ratios (OR) with 95% CI. Given the predominance of low-grade events in this cohort and the limited number of patients, regression analyses were considered exploratory and hypothesis-generating, with no formal multivariable model attempted in order to avoid overfitting. No formal a priori sample size calculation was performed, given the rarity of the event and the descriptive nature of the study; the achieved sample reflects all consecutive eligible patients within the study period.

There were no missing data for the variables of interest. A two-sided *p*-value < 0.05 was considered statistically significant. All analyses were performed using Python 3.12 with the libraries NumPy 2.0, SciPy 1.13, scikit-learn 1.5, statsmodels 0.14, and pandas 2.2.

## 3. Results

### 3.1. Patient Population

Thirty-three consecutive patients met the inclusion criteria during the 36-month study period. Mean age at the time of arthroplasty was 76.3 ± 6.3 years (range 66–85); the cohort showed a moderate female predominance (*n* = 18, 54.5%) and a mean BMI of 29.4 ± 2.8 kg/m^2^. Cardiovascular comorbidity burden was substantial: 27 patients (81.8%) had two or more relevant comorbidities, and the mean number of comorbidities per patient was 2.15 ± 0.71. Diabetes mellitus was the most prevalent (*n* = 17, 51.5%), followed by coronary artery disease (*n* = 14, 42.4%), chronic kidney disease (*n* = 13, 39.4%), atrial fibrillation (*n* = 10, 30.3%), active smoking (*n* = 9, 27.3%), and arterial hypertension (*n* = 8, 24.2%). Detailed baseline characteristics are reported in Table 1.

### 3.2. Index Orthopaedic Procedure and Vascular Injury Pattern

Twenty patients (60.6%) underwent total knee arthroplasty and 13 (39.4%) total hip arthroplasty; the right side was operated on in 19 cases (57.6%). Vascular injury was mixed arteriovenous in 18 patients (54.5%), purely venous in eight (24.2%), and purely arterial in seven (21.2%). Occlusion was the dominant lesion type (22/33, 66.7%), followed by laceration (*n* = 5), compression (*n* = 4), and compression with kinking (*n* = 2). The vessels most frequently involved were the popliteal artery (*n* = 8, 24.2%), the iliac axis including common and external iliac arteries (*n* = 6, 18.2%), the superficial femoral artery (*n* = 6, 18.2%), the common femoral artery (*n* = 5, 15.2%), the femoral vein (*n* = 5, 15.2%), and the iliac veins (*n* = 3, 9.1%).

Retractor traction was the most frequent mechanism of injury (*n* = 16, 48.5%). Direct laceration and instrumentation-related trauma were equally represented (*n* = 6 each, 18.2%), and clamp-related injuries occurred in five patients (15.2%). Underlying pathophysiology was thrombotic in 24 cases (72.7%), embolic in six (18.2%), and characterised by active bleeding in three (9.1%). Detailed lesion characteristics, time intervals, and Rutherford class at vascular consultation are reported in Table 2.

### 3.3. Time Intervals and Severity at Consultation

Mean call-to-incision time was 45.4 ± 11.3 min (median 45, IQR 38–54, range 25–65). The activation protocol was respected in all cases, with no patient exceeding 65 min between vascular team alert and operative incision. The Shapiro–Wilk test was consistent with a near-normal distribution for both call-to-incision (*p* = 0.50) and ischemia time (*p* = 0.50), supporting the use of mean ± standard deviation as descriptive summaries; comparative inference was nonetheless based on non-parametric tests, as pre-specified.

Among the 25 patients with arterial or mixed injury, the mean intraoperative ischemia time was 130.4 ± 18.7 min (median 130, IQR 120–144, range 95–160). At vascular consultation, 14 ischemic patients (56.0%) presented with Rutherford class IIa and 11 (44.0%) with class IIb. No patient presented with Rutherford class III ischemia, consistent with the rapid intraoperative recognition of the injury enabled by the on-call protocol.

### 3.4. Vascular Treatment

Treatment strategy was hybrid in 12 cases (36.4%), open surgical in 12 (36.4%), and purely endovascular in nine (27.3%). Surgical or catheter thrombectomy was the most frequent primary procedure (*n* = 20, 60.6%); primary stent placement, including covered stents in popliteal and pseudoaneurysmal lesions, was performed in nine cases (27.3%); direct venous repair (lateral suture or patch) was used in three patients; and one patient received primary arterial repair after laceration. Adjunctive procedures comprised covered or bare-metal stenting in 10 patients (30.3%), percutaneous transluminal angioplasty in seven (21.2%), and patch plasty in one (3.0%). Treatment details are summarised in Table 3.

### 3.5. Short-Term Outcomes

Technical success was achieved in all 33 patients (100%; 95% CI 89.6–100). Primary limb salvage at 30 days was likewise 100% (95% CI 89.6–100), with no major amputation. Mean postoperative ABI in arterial and mixed cases (*n* = 25) was 0.89 ± 0.03, consistent with restored, although not necessarily fully normalised, lower-limb perfusion. Median length of in-hospital stay was 11 days (IQR 8–13, range 7–14). Nineteen patients (57.6%) required intensive-care admission, with a mean ICU stay of 0.97 ± 0.92 days.

Primary 30-day patency in arterial procedures was 22/25 (88.0%; 95% CI 70.0–95.8); secondary patency reached 100% (25/25) after three reinterventions, comprising early surgical thrombectomy in two patients and stent extension in one. When stratified by treatment strategy, primary patency was 7/7 (100%) for the purely endovascular approach, 7/8 (87.5%) for hybrid procedures, and 8/10 (80.0%) for open repairs. Although the small subgroup sizes preclude formal statistical comparison (Fisher’s exact test, *p* = 0.49 for endovascular vs. open), this gradient is consistent with previous reports favouring endovascular techniques in an already-violated knee or hip operative field [18,19].

There were no 30-day deaths (0/33; 95% CI 0–10.4). Postoperative complications were entirely confined to Clavien–Dindo grade 1 events: minor self-limited bleeding (*n* = 11, 33.3%), lymphocele requiring no intervention (*n* = 11, 33.3%), and superficial wound infection treated with oral antibiotics (*n* = 5, 15.2%). Six patients (18.2%) had an entirely uneventful postoperative course, while no patient experienced a Clavien–Dindo grade ≥ 2 complication. The complete outcome panel is reported in Table 3.

### 3.6. Ischemia Time and Rutherford Class

Patients presenting with Rutherford IIb at vascular consultation had a significantly longer intraoperative ischemia time than those graded as IIa (median 144 [IQR 132–155.5] vs. 124.5 [IQR 108–129.75] minutes; mean 142.8 ± 14.2 vs. 120.6 ± 16.0 min; Mann–Whitney U = 23, *p* = 0.003; rank–biserial correlation r = 0.70, indicating a large effect size).

**Figure 1:**
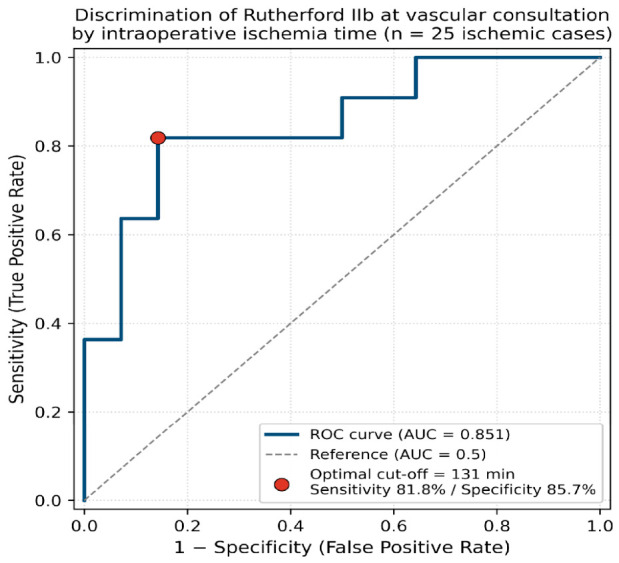
Receiver operating characteristic (ROC) curve for the discrimination of a Rutherford IIb presentation at vascular consultation by intraoperative ischemia time, in the subset of arterial and mixed cases (*n* = 25). Area under the curve (AUC) = 0.851 (95% bootstrap CI 0.679–0.982). The optimal cut-off identified by the Youden index is 131 min (sensitivity 81.8%, specificity 85.7%; Youden J = 0.675).

ROC analysis confirmed the discriminative role of ischemia time for a Rutherford IIb presentation, with an AUC of 0.851 (95% CI 0.679–0.982; *p* < 0.001 against the null hypothesis AUC = 0.5). The optimal cut-off identified by the Youden index was 131 min, providing a sensitivity of 81.8% and a specificity of 85.7% for the identification of Rutherford IIb (Youden index J = 0.675). Above this threshold, nine of 11 patients (81.8%) with prolonged intraoperative ischemia were classified as Rutherford IIb at vascular consultation, compared with two of 14 patients (14.3%) revascularized within 131 min. The corresponding ROC curve is shown in Figure 1.

### 3.7. Predictors of Postoperative Complications

Among the 33 patients, 27 (81.8%; 95% CI 65.6–91.4) developed at least one postoperative event, all graded as Clavien–Dindo 1 (lymphocele, minor self-limited bleeding, or superficial wound infection); no patient experienced a Clavien–Dindo ≥ 2 complication. The pre-specified univariate logistic regression for any postoperative event, including the seven candidate predictors listed in Section 2.7, is reported in full in Appendix A Table A1. No candidate predictor reached statistical significance at the conventional α = 0.05 level, and the diabetes mellitus coefficient did not converge because of quasi-complete separation. Given the very high baseline event rate (27/33), the small number of non-events (*n* = 6), and the homogeneous Clavien–Dindo 1 grading of all observed complications, the outcome distribution and event count preclude meaningful inference; no estimate from this exploratory analysis should be interpreted as evidence of absence of effect. An additional post hoc analysis restricted to a more stringent composite outcome (reintervention, loss of primary patency, or wound infection; *n* = 7) is reported in Appendix A Table A1 for completeness and was likewise non-informative given the small number of events.

## 4. Discussion

This single-centre cohort describes 33 consecutive iatrogenic vascular emergencies arising during elective TKA and THA and managed by a 24 h on-call vascular service. Three findings deserve emphasis. First, technical success and 30-day limb salvage were complete (100%; 95% CI 89.6–100), with primary 30-day patency of 88% and secondary patency of 100%, in a population whose average age was 76 years and in which more than four out of five patients carried at least two cardiovascular comorbidities. Second, no patient experienced a Clavien–Dindo grade ≥ 2 complication, and there were no 30-day deaths, despite the considerable surgical insult of an additional vascular access on a recently violated arthroplasty field. Third, a candidate intraoperative ischemia threshold of 131 min discriminated Rutherford IIa from IIb presentations with an AUC of 0.851, a sensitivity of 81.8%, and a specificity of 85.7%.

These limb-salvage figures sit at the upper end of contemporary expectations. Historical case series of iatrogenic arthroplasty-related injuries reported amputation rates of approximately 20% and mortality between 5 and 7%, dominated by delayed referral and late diagnosis [3,6,7]. The most recent endovascular series have substantially improved on these figures, but most still combine intra- and postoperative diagnoses and rely on inter-hospital transfer for definitive treatment [5,9,19]. Three structural factors are likely to underpin the present results: rapid intraoperative recognition by an experienced orthopaedic team, single-call activation of an institutional vascular service, in-hospital during working hours and continuously reachable through a formal on-call rota out of hours, operating in a hybrid theatre on the same campus, and consultant-level decision-making within the same operating session. Each of these elements is consistent with the explicit recommendation of the 2025 ESVS Vascular Trauma guideline that high-volume orthopaedic centres should host a continuously available 24 h vascular service capable of both open and endovascular repair [12].

The candidate’s ischemia threshold of around 131 min deserves separate consideration. The classical “six-hour rule” for irreversible muscle injury [10,20] reflects systemic, conscious ischemia in the prehospital setting and is unlikely to translate verbatim to anaesthetised, supine, often haemodynamically supported patients with a tourniquet in place or with a progressive intraoperative thrombotic occlusion. Our data suggest that, in this specific scenario, the transition between Rutherford IIa and IIb occurs substantially earlier, broadly between two and two and a half hours of intraoperative ischemia. Several mechanisms can plausibly compress this time window in arthroplasty patients: distal embolisation from the manipulation of calcified iliofemoral and popliteal vessels, propagation of secondary thrombus during retractor-related stasis, reduced metabolic reserve in elderly multimorbid muscle, and the additive effect of tourniquet ischemia at the knee [3,8,20]. Although a 25-patient subset cannot establish causality, the convergence of a significant difference in median ischemia times (144 vs. 124.5 min; *p* = 0.003), a large rank–biserial effect size (r = 0.70), and a robust ROC AUC with a lower bootstrap bound of 0.679 lends internal coherence to the threshold and makes it a credible target for prospective validation. We acknowledge the constraint imposed by sample size, which reflects the rarity of the event rather than a methodological choice; all consecutive eligible patients over 36 months were included. The statistical framework was selected to be appropriate for the subset size: the Hanley–McNeil-based standard error for the AUC, combined with 2000-iteration bootstrap resampling for the confidence interval, represents the reference method for diagnostic accuracy studies in small, population-constrained cohorts. The lower bound of the bootstrap AUC CI (0.679) approaches the commonly accepted threshold of 0.70 for acceptable discriminative performance. Taken together, the multi-evidence convergence outlined above supports the threshold as a biologically plausible and statistically coherent hypothesis, which we explicitly frame as requiring prospective validation in larger multicentre registries before clinical adoption.

Operationally, a 131 min mark would not replace the cardinal principle of immediate vascular activation at the very first sign of distal pulse loss [11,12]; rather, it provides a quantitative reminder that a non-trivial proportion of patients progress to an advanced ischemic class within a relatively narrow time window, well before the six-hour limit usually invoked in non-iatrogenic ALI. In our protocol, the mean call-to-incision time was 45 min and never exceeded 65; further compression of this interval is probably not feasible in practice and may not be necessary, but the data argue against any tolerance for waiting and against the temptation to defer activation in apparently mild cases of pulse loss “until after the cementing” or similar intraoperative milestones.

With respect to revascularization strategy, our experience matches the gradient observed in the current literature. Open repair retains a clear role for direct laceration, transection, and lesions requiring large-vessel reconstruction, particularly when intrapelvic exposure is necessary at the hip [6,13]. The endovascular and hybrid options have, however, progressively gained ground in the already-violated arthroplasty field, where re-entering the same access for an open exploration adds both haemorrhagic and infective risk to the burden of an additional surgical insult [4,8,18]. The numerically higher primary 30-day patency observed for purely endovascular cases (7/7) compared with hybrid (7/8) and open (8/10) repair did not reach statistical significance and must be read with caution given the small subgroups, but is fully concordant with the algorithm proposed by Gosslau et al. [8] and with the systematic review by Melian et al. [21], which both endorse an endovascular-first strategy when anatomy and lesion morphology permit. Retractor traction was the dominant mechanism (48.5%), again consistent with previous series for both hip and knee [3,6,8], and reinforces the value of periodic intraoperative pulse and continuous-wave Doppler checks, especially in elderly patients with calcified vessels.

The high overall rate of postoperative events (81.8%) requires careful reading. Every event was graded Clavien–Dindo 1: lymphocele requiring no intervention, minor self-limited bleeding, or superficial wound infection treated with oral antibiotics, and no patient progressed to a grade ≥ 2 complication. Such a profile is largely the expected consequence of a vascular access added to a recently operated joint in a population whose mean age exceeds 75 years and whose comorbidity burden includes diabetes mellitus in roughly half of cases. To clarify the clinical significance of these events, the complication profile was disaggregated to separate clinically meaningful endpoints from minor, self-limiting sequelae. Among the 27 patients with any grade 1 complication, the three constituent subtypes were: minor self-limited bleeding managed conservatively without transfusion or reintervention (*n* = 11, 33.3%); lymphocele requiring no procedural intervention (*n* = 11, 33.3%); and superficial wound infection treated with a standard oral antibiotic course (*n* = 5, 15.2%). None of these events required reoperation, radiological drainage, blood transfusion, or prolonged hospitalisation. The post hoc composite of clinically meaningful endpoints, including reintervention, loss of primary patency, or wound infection, occurred in seven patients (21.2%); this analysis was likewise non-informative due to the small event count, consistent with the exploratory framing. Zero patients experienced a Clavien–Dindo ≥ 2 event, precluding any stratum-specific comparison with higher-grade complications. The Clavien–Dindo classification has been formally validated in arthroplasty populations and shown to correlate meaningfully with discharge disposition and length of stay in this specific surgical context [22]. The absence of any predictor reaching statistical significance in the exploratory univariate regression (Appendix A Table A1) reflects the truncated outcome distribution and the limited number of patients without complications (*n* = 6) rather than a true absence of risk gradient: the high baseline rate of minor events compresses the dynamic range available for inference, and quasi-complete separation made the diabetes coefficient inestimable. Consistent with this, and with the pre-specification, all regression analyses are framed as exploratory, and no individual coefficient is interpreted as causal or prognostic.

Beyond their immediate clinical interest, these data carry an organisational message. With TKA volumes projected to approach 1.2 million procedures per year by 2040 in the United States alone [1,2], the absolute number of iatrogenic vascular injuries will inevitably grow even if the relative incidence remains stable. In this perspective, the present cohort supports the structural argument that high-volume orthopaedic centres should host a dedicated, single-institution vascular service, continuously available through in-hospital daytime coverage and a formal out-of-hours on-call rota, rather than rely on inter-hospital transfer. The inclusion of an objective intraoperative metric, a candidate threshold close to 130 min of ischemia, in local activation algorithms could complement the established clinical triggers and provide a quantitative reference for second-tier escalation when the lesion mechanism, vessel involved, or anaesthetic constraints make immediate intraoperative reassessment more difficult.

The principal strength of this study lies in the homogeneity of the setting. All 33 patients were managed under a single, prospectively defined on-call protocol, in the same hybrid theatre, by the same vascular team, over a contiguous 36-month period, with complete 30-day follow-up. This minimises pathway-related heterogeneity that has historically confounded the iatrogenic vascular injury literature [3,5,13]. Several limitations should be acknowledged. The retrospective single-centre design and the relatively small sample size, although consistent with the rarity of the event, restrict statistical power and external generalisability. The absence of a contemporaneous control group of patients managed in centres without a dedicated institutional vascular service prevents a direct comparison of pathways. A randomised design or concurrent control arm is not feasible in this emergency setting: the absolute incidence of vascular injury during arthroplasty (0.05–0.3%) would require several thousand procedures per arm, and it would be ethically impermissible to withhold immediate vascular access once injury is identified. We therefore contextualise our outcomes against the published literature from centres that relied on inter-hospital transfer for definitive vascular repair, where amputation rates of approximately 20–21% and mortality of 5–7% are consistently reported [3,6,7]. The favourable results in the present cohort are plausibly attributable to a combination of structural factors: rapid intraoperative recognition by an experienced orthopaedic team, single-call activation of a consultant vascular service operating in a hybrid theatre on the same campus, and decision-making within the same operative session, each of which is consistent with the 2025 ESVS Vascular Trauma guideline recommendation [12]. We acknowledge, however, that early recognition skills, surgeon expertise, and referral selection bias cannot be formally disentangled from the organisational model in a retrospective single-centre series. Follow-up was deliberately limited to 30 days, which leaves longer-term patency, residual claudication, and walking recovery unaddressed; longer-term ABI may also be more informative than its postoperative measurement, particularly in elderly diabetic patients in whom resting ABI has well-known limitations [18]. The 131 min threshold is derived from a single 25-patient subset and should be regarded as a hypothesis for prospective validation in larger multicentric registries, ideally including patient-level data on patency and functional outcomes at one year and beyond. Finally, the regression analyses are explicitly exploratory, are not corrected for multiplicity, and do not support any causal inference.

Future research could usefully focus on three lines: prospective multicentric validation of the proposed ischemia threshold and of the on-call activation pathway; the role of objective intraoperative monitoring tools, continuous-wave Doppler, near-infrared spectroscopy, or intraoperative duplex, as automated triggers for vascular activation; and longer-term functional and quality-of-life outcomes after iatrogenic arthroplasty-related vascular repair, an area still largely undocumented in the available literature [5,9,19].

## 5. Conclusions

An intraoperative ischemia time of approximately 131 min identifies the transition from Rutherford IIa to IIb at vascular consultation (AUC 0.851) and may serve, after prospective validation, as a candidate operational marker for early multidisciplinary activation in high-volume orthopaedic centres. In the context of a tertiary orthopaedic referral centre with a dedicated 24 h vascular service, emergency repair of iatrogenic vascular injuries occurring during elective TKA or THA achieved 100% technical success and 30-day limb salvage, primary 30-day patency of 88%, secondary patency of 100%, and no Clavien–Dindo grade ≥ 2 events or 30-day mortality, outcomes that confirm the validity of the on-call model within which the threshold was derived. Within the structural framework recommended by the 2025 ESVS Vascular Trauma guideline, these findings lend pragmatic support to the maintenance of a single-institution vascular service, continuously available through structured daytime coverage and an out-of-hours on-call rota, in high-volume orthopaedic centres.

## Figures and Tables

**Table 1:** Baseline patient characteristics.

Variable	Value	%/Range
Number of patients	33	—
Age, mean ± SD (years)	76.3 ± 6.3	66–85
Female sex, *n* (%)	18	54.5%
BMI, mean ± SD (kg/m^2^)	29.4 ± 2.8	24–34
Diabetes mellitus, *n* (%)	17	51.5%
Coronary artery disease, *n* (%)	14	42.4%
Chronic kidney disease, *n* (%)	13	39.4%
Atrial fibrillation, *n* (%)	10	30.3%
Active smoking, *n* (%)	9	27.3%
Arterial hypertension, *n* (%)	8	24.2%
≥2 comorbidities, *n* (%)	27	81.8%
Mean number of comorbidities ± SD	2.15 ± 0.71	1–3
Index procedure: TKA, *n* (%)	20	60.6%
Index procedure: THA, *n* (%)	13	39.4%
Right side, *n* (%)	19	57.6%

**Table 2:** Vascular injury characteristics, time intervals, and Rutherford class at vascular consultation.

Variable	Value	%/Range
Injury type, combined arteriovenous, *n* (%)	18	54.5%
Injury type, venous, *n* (%)	8	24.2%
Injury type, arterial, *n* (%)	7	21.2%
Pattern, occlusion, *n* (%)	22	66.7%
Pattern, laceration, *n* (%)	5	15.2%
Pattern, compression, *n* (%)	4	12.1%
Pattern, compression/kinking, *n* (%)	2	6.1%
Vessel, popliteal artery, *n* (%)	8	24.2%
Vessel, iliac axis, *n* (%)	6	18.2%
Vessel, superficial femoral artery, *n* (%)	6	18.2%
Vessel, common femoral artery, *n* (%)	5	15.2%
Vessel, femoral vein, *n* (%)	5	15.2%
Vessel, iliac vein, *n* (%)	3	9.1%
Mechanism, retraction, *n* (%)	16	48.5%
Mechanism, direct injury, *n* (%)	6	18.2%
Mechanism, instrumentation, *n* (%)	6	18.2%
Mechanism, clamp-related, *n* (%)	5	15.2%
Pathophysiology, thrombosis, *n* (%)	24	72.7%
Pathophysiology, embolism, *n* (%)	6	18.2%
Pathophysiology, bleeding, *n* (%)	3	9.1%
Call-to-incision time, mean ± SD (min)	45.4 ± 11.3	25–65
Call-to-incision time, median (IQR) (min)	45 (38–54)	—
Ischemia time (*n* = 25), mean ± SD (min)	130.4 ± 18.7	95–160
Ischemia time (*n* = 25), median (IQR) (min)	130 (120–144)	—
Rutherford IIa (of 25 ischemic), *n* (%)	14	56.0%
Rutherford IIb (of 25 ischemic), *n* (%)	11	44.0%
Rutherford III, *n* (%)	0	0.0%

**Table 3:** Treatment strategy and 30-day outcomes.

Variable	Value	% (95% CI)/Range
Strategy, open, *n* (%)	12	36.4%
Strategy, hybrid, *n* (%)	12	36.4%
Strategy, endovascular, *n* (%)	9	27.3%
Procedure, thrombectomy, *n* (%)	20	60.6%
Procedure, primary stenting, *n* (%)	9	27.3%
Procedure, venous repair, *n* (%)	3	9.1%
Procedure, primary arterial repair, *n* (%)	1	3.0%
Adjunct, stent (covered/BMS), *n* (%)	10	30.3%
Adjunct, PTA, *n* (%)	7	21.2%
Adjunct, patch plasty, *n* (%)	1	3.0%
Technical success	33/33	100% (89.6–100)
30-day limb salvage	33/33	100% (89.6–100)
Postoperative ABI (*n* = 25), mean ± SD	0.89 ± 0.03	0.85–0.96
Length of stay, median (IQR) (days)	11 (8–13)	7–14
ICU admission, *n* (%)	19	57.6%
ICU stay, mean ± SD (days)	0.97 ± 0.92	0–2
Primary 30-day patency (arterial)	22/25	88% (70.0–95.8)
Secondary 30-day patency	25/25	100%
Reintervention, *n* (%)	3	9.1% (3.1–23.6)
Clavien–Dindo 0, *n* (%)	6	18.2%
Clavien–Dindo 1, *n* (%)	27	81.8% (65.6–91.4)
Clavien–Dindo ≥ 2, *n* (%)	0	0%
30-day mortality, *n* (%)	0	0% (0–10.4)

## Data Availability

The datasets analysed during the current study are not publicly available because of institutional restrictions on anonymised clinical data; anonymised aggregate data may be available from the corresponding author on reasonable request.

## Supplementary Materials

The following supporting information can be downloaded at: https://www.mdpi.com/article/10.3390/jcm15135229/s1, Table S1: Strobe Checklist.

## Abbreviations

The following abbreviations are used in this manuscript:

ABI	ankle–brachial index
ALI	acute limb ischemia
AUC	area under the curve
BMI	body mass index
BMS	bare-metal stent
CI	confidence interval
ESVS	European Society for Vascular Surgery
ICU	intensive-care unit
IQR	interquartile range
OR	odds ratio
PTA	percutaneous transluminal angioplasty
ROC	receiver operating characteristic
SD	standard deviation
STROBE	Strengthening the Reporting of Observational Studies in Epidemiology
THA	total hip arthroplasty
TKA	total knee arthroplasty

## Appendix A

**Table A1:** Pre-specified exploratory univariate logistic regression for any postoperative complication (Clavien–Dindo grade ≥ 1) and post hoc univariate analysis for the stringent composite outcome (reintervention, loss of primary patency, or wound infection). Estimates are reported for completeness only: the high baseline event rate (27/33 for the primary composite) and the small number of non-events preclude meaningful inference, and no individual coefficient should be read as evidence of absence of effect.

Predictor	OR	95% CI	*p*
Panel A Any postoperative complication (Clavien–Dindo grade ≥ 1; 27/33 events)			
Ischemia time (per minute) †	0.99	0.93–1.06	0.872
Rutherford IIb (vs IIa) †	1.67	0.13–21.20	0.694
Call-to-incision time (per minute)	1.02	0.94–1.10	0.670
Age (per year)	0.82	0.66–1.02	0.078
BMI (per unit)	0.88	0.63–1.23	0.452
Male sex	1.86	0.29–11.90	0.514
Diabetes mellitus	—	—	n.c. ‡
Panel B Stringent composite outcome (reintervention, loss of primary patency, or wound infection; 7/33 events; post hoc) †			
Ischemia time (per minute)	1.02	0.97–1.07	0.53
Rutherford IIb (vs IIa)	0.56	0.08–3.80	0.55

† Estimated in the subset of arterial/mixed cases (*n* = 25). ‡ Coefficient did not converge because of quasi-complete separation. OR, odds ratio; CI, confidence interval; n.c., not converged.

## References

[1] Sloan M., Premkumar A., Sheth N.P. (2018). Projected Volume of Primary Total Joint Arthroplasty in the U.S., 2014 to 2030. J. Bone Jt. Surg..

[2] Singh J.A., Yu S., Chen L., Cleveland J.D. (2019). Rates of Total Joint Replacement in the United States: Future Projections to 2020–2040 Using the National Inpatient Sample. J. Rheumatol..

[3] Sundaram K., Udo-Inyang I., Mont M.A., Molloy R., Higuera-Rueda C., Piuzzi N.S. (2020). Vascular Injuries in Total Knee Arthroplasty: A Systematic Review and Meta-Analysis. JBJS Rev..

[4] Galassi L., Santi G., Mercandalli G., Ravini M.L., Cugliari M. (2025). Delayed-Onset Pseudoaneurysm of the Superior Lateral Genicular Artery Following Total Knee Arthroplasty: A Case Report. Clin. Med. Insights Case Rep..

[5] Hodgson H., Saghir N., Saghir R., Coughlin P., Scott D.J.A., Howard A. (2023). Arterial Complications Following Total Knee Arthroplasty (TKA): A Systematic Review and Proposal for Improved Monitoring. Malays. Orthop. J..

[6] Alshameeri Z., Bajekal R., Varty K., Khanduja V. (2015). Iatrogenic Vascular Injuries during Arthroplasty of the Hip. Bone Jt. J..

[7] Bernhoff K., Rudström H., Gedeborg R., Björck M. (2013). Popliteal Artery Injury during Knee Replacement: A Population-Based Nationwide Study. Bone Jt. J..

[8] Gosslau Y., Warm T.D., Foerch S., Zerwes S., Scheurig-Muenkler C., Hyhlik-Duerr A. (2022). Iatrogenic Injury of the Popliteal Artery in Orthopedic Knee Surgery: Clinical Results and Development of a Therapeutic Algorithm. Eur. J. Trauma Emerg. Surg..

[9] Barabino E., Pittaluga G., Nivolli A., Ivaldi D., Arnò M., Gazzo P. (2023). Endovascular Management of Iatrogenic Arterial Injuries after Orthopedic Surgery of the Lower Limb. J. Vasc. Interv. Radiol..

[10] Olinic D.-M., Stanek A., Tătaru D.-A., Homorodean C., Olinic M. (2019). Acute Limb Ischemia: An Update on Diagnosis and Management. J. Clin. Med..

[11] Björck M., Earnshaw J.J., Acosta S., Bastos Gonçalves F., Cochennec F., Debus E.S., Hinchliffe R., Jongkind V., Koelemay M.J.W., Menyhei G. (2020). Editor’s Choice—European Society for Vascular Surgery (ESVS) 2020 Clinical Practice Guidelines on the Management of Acute Limb Ischaemia. Eur. J. Vasc. Endovasc. Surg..

[12] Wahlgren C.M., Aylwin C., Davenport R.A., Davidovic L.B., DuBose J.J., Gaarder C., Heim C., Jongkind V., Jørgensen J., Kakkos S.K. (2025). Editor’s Choice—European Society for Vascular Surgery (ESVS) 2025 Clinical Practice Guidelines on the Management of Vascular Trauma. Eur. J. Vasc. Endovasc. Surg..

[13] Eliason J.L., Wainess R.M., Proctor M.C., Dimick J.B., Cowan J.A., Upchurch G.R., Stanley J.C., Henke P.K. (2003). A National and Single Institutional Experience in the Contemporary Treatment of Acute Lower Extremity Ischemia. Ann. Surg..

[14] Von Elm E., Altman D.G., Egger M., Pocock S.J., Gøtzsche P.C., Vandenbroucke J.P. (2014). The Strengthening the Reporting of Observational Studies in Epidemiology (STROBE) Statement: Guidelines for Reporting Observational Studies. Int. J. Surg..

[15] Rutherford R.B., Baker J.D., Ernst C., Johnston K.W., Porter J.M., Ahn S., Jones D.N. (1997). Recommended Standards for Reports Dealing with Lower Extremity Ischemia: Revised Version. J. Vasc. Surg..

[16] Dindo D., Demartines N., Clavien P.-A. (2004). Classification of Surgical Complications: A New Proposal With Evaluation in a Cohort of 6336 Patients and Results of a Survey. Ann. Surg..

[17] Clavien P.A., Barkun J., De Oliveira M.L., Vauthey J.N., Dindo D., Schulick R.D., De Santibañes E., Pekolj J., Slankamenac K., Bassi C. (2009). The Clavien-Dindo Classification of Surgical Complications: Five-Year Experience. Ann. Surg..

[18] AbuRahma A.F., Adams E., AbuRahma J., Mata L.A., Dean L.S., Caron C., Sloan J. (2020). Critical Analysis and Limitations of Resting Ankle-Brachial Index in the Diagnosis of Symptomatic Peripheral Arterial Disease Patients and the Role of Diabetes Mellitus and Chronic Kidney Disease. J. Vasc. Surg..

[19] Tsouknidas I., Mendez A., Kats A., Hirsch H., Meisner R., Hayes D., Patton G., Uribe A., DiGiovanni V. (2024). Outcomes of Endovascular Treatment for Popliteal Artery Injury After Total Knee Arthroplasty. J. Vasc. Surg..

[20] Galassi L., Ravini M.L., Bassani R., Mercandalli G., Santoro G.D. (2025). In the Shadow of Stability Lies Ruin: Occult Vascular Injuries in Geriatric Pelvic Trauma. World J. Clin. Cases.

[21] Melian C.M., Giannopoulos S., Tsouknidas I., Volteas P., Virvilis D., Nicholson J., Koullias G.J. (2023). Endovascular Repair of Popliteal Artery Injury Post-Total Knee Arthroplasty Is Safe and Effective: A Case Report and Systematic Review of the Literature. Ann. Vasc. Surg..

[22] Rele S., Shadbolt C., Schilling C., Thuraisingam S., Trieu J., Choong E.L.P., Gould D., Taylor N.F., Dowsey M.M., Choong P.F.M. (2025). Validation of the Clavien-Dindo Classification and Comprehensive Complication Index as Measures of Morbidity Following Total Hip and Knee Arthroplasty. Bone Jt. J..

